# Symptom Management and Support in Dying Patients with Cancer and Coronavirus Disease-19—A Register-Based Study

**DOI:** 10.1177/08258597231157622

**Published:** 2023-02-15

**Authors:** Christel Hedman, Peter Strang, Staffan Lundström, Lisa Martinsson

**Affiliations:** 1 Department of Molecular Medicine and Surgery, 27106 Karolinska Institutet, Stockholm, Sweden; 2 R&D Department, Stockholms Sjukhem Foundation, Stockholm, Sweden; 3 Department of Clinical Sciences Lund, Lund University, Lund, Sweden; 4 Department of Oncology–Pathology, 27106 Karolinska Institutet, Stockholm, Sweden; 5 Department of Radiation Sciences, 8075 Umeå University, Umeå, Sweden

**Keywords:** COVID-19, palliative care, end-of-life care, cancer, symptoms, hospital care

## Abstract

**Objective:**

Little is known to what extent access to specialist palliative care (SPC) for cancer patients dying with coronavirus disease-2019 (COVID-19) affects the occurrence of breakthrough symptoms, symptom relief, and overall care, compared to hospital deaths. Our aim was to include patients with both COVID-19 and cancer and compare those dying in hospitals with those dying in SPC with reference to the quality of end-of-life care.

**Methods:**

Patients with both cancer and COVID-19 who died in hospitals (*n* = 430) and within SPC (*n* = 384) were identified from the Swedish Register of Palliative Care. The hospital and SPC groups were compared regarding the quality of end-of-life care, including the occurrence of 6 breakthrough symptoms during the last week in life, symptom relief, end-of-life care decisions, information, support, and human presence at death.

**Results:**

Breakthrough of breathlessness was more common in the hospital patients compared to the SPC patients (61% and 39%, respectively; *p* < .001), while pain was less common (65% and 78%, respectively; *p* < .001). Breakthrough of nausea, anxiety, respiratory secretions, or confusion did not differ. All 6 symptoms, except for confusion, were more often completely relieved in SPC (*p* = .014 to *p* < .001 in different comparisons). In SPC, a documented decision about the goal being end-of-life care and information about this were more common than in hospitals (*p* < .001). Also, to have family members present at the time of death and for family members to be offered a follow-up talk afterward was more common in SPC (*p* < .001).

**Conclusion:**

More systematic palliative care routines may be an important factor for better symptom control and higher quality of end-of-life care in hospitals.

## Introduction

The coronavirus disease-2019 (COVID-19) pandemic has seriously affected the whole world since it started at the beginning of 2020. Patients with cancer have been affected by the COVID-19 pandemic in numerous ways. Those with active cancer or ongoing cancer treatment have an increased risk of getting a COVID-19 infection, an elevated risk of severe disease, and, in addition, higher mortality from COVID-19.^1–3^ Furthermore, an increase in avoidable deaths due to diagnostic delays of cancer is to be expected.^4^

Previous pandemics have shown a profound effect on end-of-life (EOL) care due to a reduction of palliative care services; difficulties in allowing a family to be present at EOL due to visiting restrictions; a shortage of medications, trained healthcare workers with knowledge about symptom management, and protective equipment.^5^ During the COVID-19 pandemic, visiting restrictions have had a major impact on EOL care with increasing loneliness, the risk of aggravated symptoms, and being alone during the dying phase.^6–8^ Moreover, relatives have not had the possibility to say goodbye or to communicate properly with either the patients or the healthcare professionals (HCPs)^9^ possibly affecting the bereavement process. The above-mentioned findings are extracted from general studies, whereas studies focusing on cancer patients dying from COVID-19 are scarce.

Studies have shown that the symptom burden in those dying from COVID-19 is partly different from that of patients dying from other diagnoses, with more difficulties with breathlessness, delirium, and anxiety.^6,10,11^ Symptom relief is one of the cornerstones of palliative care,^12^ as patients in the EOL often experience a variety of symptoms affecting their well-being and quality of life.^13,14^ Specialist palliative care (SPC) has been shown to improve symptom control in patients in the EOL, both in cancer and noncancer settings.^15,16^

Despite this, little is known about how integration of a palliative care approach into COVID-19 care can affect symptom relief and support to relatives of cancer patients with COVID-19.^17^ As patients with advanced cancer often need SPC, those cancer patients with COVID-19 might even have more pronounced need. However, these patients seemed to have less possibility of being referred to SPC compared to those without a COVID-19 infection, at least during the first wave.^18^

Only 1 study assessed patients with cancer and COVID-19 during EOL care, showing that these patients had a high symptom burden.^19^ Thus, to maintain high-quality palliative care, structured assessments of pain and other symptoms are considered essential.^20^ Unfortunately, adherence to these clinical routines has not met the usual standard during the COVID-19 pandemic,^21^ and there are no studies addressing clinical routines in cancer patients during the pandemic. In addition, there is little knowledge of how palliative care can contribute to the care of dying patients with cancer and COVID-19.

The aim was to study patients with concomitant COVID-19 and cancer and compare those dying in hospitals with those dying in SPC services, with reference to symptoms and symptom control, the possibility for relatives to be present, communication offered by HCPs and adherence to clinical routines during EOL care.

## Methods

### Study Design

The data in this study were registered in a national quality register collecting data about EOL care, the Swedish Register of Palliative Care (SRPC). The SRPC database has been used by the research group in previous studies, and data collection methods have been described earlier.^6,21^ The information in the SRPC is based on data registered by HCPs about individuals who have died, focusing on symptoms and other important EOL care issues during the last week in life. Data were first retrieved by the research group from the SRPC database on 7 September 2021 and additional data were retrieved on 9 May 2022.

### Setting and Participants

All adult patients with cancer as 1 of the main diagnoses causing death and a subsequent COVID-19 diagnosis who had died before 7 September 2021 were reported from acute hospitals and SPC to the SRPC were included. No information on the severity of the COVID-19 infection or the stage of the malignancy is available from the SRPC. In Sweden, SPC is offered both inpatient specialized care and specialized palliative home care. Inpatient specialized care mostly takes care of patients during the last weeks of life, while those in specialized advanced home care can be included for a longer period.

Each year, around 60% of all deaths in Sweden are reported to the SRPC,^22^ and for cancer diagnoses the corresponding figure is 80%.^23^

On 8 February 2021, the SRPC changed the way they collected data about COVID-19 infections. For those who died after 8 February 2021, the SRPC specifies how many days before death the COVID-19 diagnosis was established. For this study, we chose only those who had died within 30 days of their COVID-19 diagnosis among those for whom this information was available.

Data are lacking about the quality of EOL care for patients who were reported by the HCP to have died unexpectedly according to the disease trajectory, since these data, according to the routines of the SRPC, are not registered for unexpected deaths. Consequently, patients who died an unexpected death were not included in the analyses.

The analyses included the following items from the SRPC database: whether assessments of oral health, pain, and other symptoms were performed during the last week in life, occurrence of 6 breakthrough symptoms, and relief of these symptoms during the last week of life. Moreover, EOL care decisions, information to the patient and family, whether someone was present at the time of death, whether family members were offered a follow-up talk, prescriptions of pro re nata (p.r.n.) medications for symptom alleviation and fluids/enteral nutrition during the last 24 h were analyzed. Breakthrough symptoms were defined as symptoms occurring despite possible medication for that symptom.

### Ethical Considerations

No informed consent was provided as all patients were deceased at inclusion.

### Statistical Analysis

Proportions were calculated using the χ^2^-test and ages were compared using the *t*-test. Symptom relief is reported to the SRPC as complete, partial, or no relief. Both the proportions of patients with complete or partial relief (vs. no relief) and the proportions of patients with complete relief (vs. partial or no relief) were compared between the hospital and SPC groups. Patients who were reported to have no family members or close friends were excluded from the analyses about information and support to family/close friends. Patients whose family members declined information about transition to EOL care were excluded from the analysis of that corresponding item. Most items analyzed in this study could be answered by the reporting HCP as “Don’t know in the SRPC database. These cases were excluded from further analyses of the corresponding item. Logistic regression was used to examine the impact on the place of death for the 2 most common breakthrough symptoms breathlessness and pain, adjusting for age (continuous), gender, and cardiovascular disease.

## Ethical Approval

The working procedure and study design were examined by the Ethical Review Board in Sweden, and they had no ethical objections to the study (registration number 2020-02186). The study was conducted with consent from the SRPC management group.

## Results

In total, 874 patients who died in hospitals or SPC with cancer and COVID-19 were identified in the database. From hospitals, 485 patients were identified, of whom 55 were reported as unexpected deaths and consequently excluded, resulting in 430 patients. From inpatient specialized care, 265 patients were identified, with 1 being an unexpected death. From specialized palliative home care, 124 were reported, of whom 4 were unexpected deaths, adding up to 384 patients in SPC. A total of 814 patients remained in the study (Table 1).

**Table 1.** Comparison of Sex, Age, and Comorbidities Between the Hospital Group and the SPC Group.

		Hospital patients (*n* = 430)	SPC patients (*n* = 384)	*P*-value
Men		273/430 (63%)	204/384 (53%)	.003
Age				
	Mean	77.2	74.0	< .001
	Range	31–101	31–100	—
Comorbidities contributing to death (one person could have multiple diseases reported)				
	Cardiovascular disease	122/430 (28%)	36/384 (9%)	< .001
	Dementia	25/430 (6%)	7/384 (2%)	.003
	Diabetes	44/430 (10%)	6/384 (2%)	< .001

Abbreviations: SPC, specialist palliative care.

The hospital patients were more often men, were older, and had more comorbidity compared to the SPC group; see Table 1.

Assessment of oral health, pain, and other symptoms were more commonly performed during the last week in SPC compared to hospitals. The occurrence of breakthrough breathlessness during the last week of life was more common in hospital patients (61%) compared to SPC patients (39%), while the pain was less common in hospitals (65% compared to 78% in SPC) (Table 2). In the regression model, both occurrence of breakthrough breathlessness and breakthrough pain still differed significantly between SPC and hospitals after adjusting for gender, age, and whether the patient had cardiovascular disease (Table 3). The number of patients with the breakthrough of nausea, anxiety, respiratory secretions, or confusion did not differ between the groups (Table 2).

**Table 2.** Comparison of Symptom Assessments, Occurrence of Breakthrough Symptoms and Symptom Relief Between Hospital Patients and SPC Patients.

			Hospital patients	SPC patients	*P*-value
Assessments					
Oral health assessed			220/372 (57%)	324/376 (86%)	<.001
Pain assessed			152/381 (40%)	300/377 (80%)	<.001
Other symptoms assessed			87/358 (24%)	191/368 (52%)	<.001
Breathlessness					
		Occurrence^a^	242/394 (61%)	147/376 (39%)	<.001
		Complete or partial relief^b^	227/242 (94%)	142/147 (97%)	.23
		Complete relief^c^	63/242 (26%)	80/147 (54%)	<.001
Pain					
		Occurrence^a^	258/396 (65%)	297/379 (78%)	<.001
		Complete or partial relief^b^	255/258 (99%)	297/297 (100%)	.10^d^
		Complete relief^c^	158/258 (61%)	248/297 (84%)	<.001
Nausea					
		Occurrence^a^	44/331 (13%)	52/367 (14%)	.74
		Complete or partial relief^b^	42/44 (95%)	51/52 (98%)	.59^d^
		Complete relief^c^	16/44 (36%)	38/52 (73%)	<.001
Anxiety					
		Occurrence^a^	261/369 (71%)	242/360 (67%)	.31
		Complete or partial relief^b^	256/261 (98%)	240/242 (99%)	.45^d^
		Complete relief^c^	131/261 (50%)	191/242 (79%)	<.001
Respiratory secretions					
	Occurrence^a^		190/399 (48%)	199/384 (52%)	.24
	Complete or partial relief^b^		171/190 (90%)	184/199 (92%)	.39
	Complete relief^c^		71/190 (37%)	99/199 (50%)	.014
Confusion					
	Occurrence^a^		128/345 (37%)	123/367 (34%)	.32
	Complete or partial relief^b^		103/128 (80%)	104/123 (85%)	.39
	Complete relief^c^		24/128 (19%)	35/123 (28%)	.070

Abbreviation: SPC, specialist palliative care

a “Don’t know” was an answering option for the items about symptom occurrence. Therefore, numbers do not sum to group totals.

b Complete or partial relief versus no relief. Only cases where that corresponding symptom occurred during the last week are included in the analysis.

c Complete relief versus partial or no relief. Only cases where that corresponding symptom occurred during the last week are included in the analysis.

d Chi-square calculations were substituted with Fisher exact test in this comparison, as values in 2 of the cells in the contingency table were <5.

**Table 3.** Logistic Regression Model Regarding Impact of Place of Death on Symptoms, Adjusted for Age, Gender, and Cardiovascular Disease.

	Place of death (ref: SPC)			Gender (ref: men)			Age in years (continuous)			Cardiovascular disease		
	OR	95% CI	*P*-value	OR	95% CI	*P*-value	OR	95% CI	*P*-value	OR	95% CI	*P*-value
Breathlessness occurrence	2.32	1.72–3.13	< .001	.92	.68–1.24	.58	1.00	.99–1.01	.97	1.38	.83–2.06	.11
Pain occurrence	.55	.40–.77	< .001	1.63	1.17–2.28	.004	.99	.97–1.00	.076	1.05	.69–1.58	.84

Abbreviations: SPC, specialist palliative care; OR, odds ratio; CI, confidence interval.

There were no differences between the hospital and SPC patients regarding the proportion of patients who were at least partially relieved from their symptoms. However, all 6 symptoms, except for confusion, were more often completely relieved in SPC as compared to hospitals (Table 2).

In SPC, a documented decision about the goal being EOL care and information about the transition to EOL care was more common than in hospitals. Furthermore, to have family members present at the time of death and for family members to be offered a follow-up talk afterward was more common in SPC (Table 4).

**Table 4.** Comparison of End-of-Life Care Decisions, Information, Human Presence at the Time of Death and Support to Families Between Hospital Patients and SPC Patients.^a^

		Hospital patients	SPC patients	*P*-value
Documented decision about the goal being end-of-life care		352/419 (84%)	373/382 (98%)	<.001
Information to the patient about transition to end-of-life care (including only patients who had the ability to participate in such conversation)		191/280 (68%)	333/349 (95%)	<.001
Information to the family/close friends about transition to end-of-life care^b^		329/382 (86%)	358/371 (96%)	<.001
**Present at time of death**				
	Family and/or staff	277/417 (66%)	294/382 (77%)	<.001
	Family only	111/417 (27%)	159/382 (42%)	<.001
	Staff only	120/417 (29%)	83/382 (22%)	.022
Family/close friends offered a follow-up talk^c^		199/296 (67%)	355/365 (97%)	<.001

Abbreviation: SPC, specialist palliative care.

^a^ “Don’t know” was an answering option for all items included in the table. Therefore, numbers do not sum to group totals.

^b^ Patients without family/close friends (*n* = 4 in hospitals, 3 in inpatient specialized palliative care) and patients for whom the family members declined to participate in this conversation (*n* = 3 in hospitals, 1 in inpatient specialized palliative care and 3 in specialized palliative home care) were excluded.

^c^ Patients without family/close friends (*n* = 4 in hospitals, 3 in inpatient specialized palliative care) were excluded.

Fluids/enteral tube nutrition was less frequently administered in SPC during the last 24 h of life. To have been examined by a physician during the last days of life was more common in hospitals. In the hospital group, 14% had a palliative care team consulted regarding symptom relief (not including the 49 “Don’t know” answers) (Table 5).

**Table 5.** Comparison of End-of-Life Care Routines Between Hospital Patients and SPC Patients.^a^

	Hospital patients	SPC patients	*P*-value
p.r.n. strong opioid prescribed	405/429 (94%)	381/384 (99%)	<.001^b^
p.r.n. tranquilizer prescribed	402/428 (94%)	379/384 (99%)	<.001
p.r.n. antiemetic prescribed	347/426 (81%)	372/384 (97%)	<.001
p.r.n. antimuscarinic prescribed	374/429 (87%)	370/384 (96%)	<.001
Examined by physicians during the last days before death	423/425 (99.5%)	354/384 (92%)	<.001^b^
A palliative care team was consulted	53/379 (14%)	NA^a^	
Fluids and/or enteral tube nutrition during the last 24 h	142/422 (34%)	27/384 (7%)	<.001

Abbreviations: SPC, specialist palliative care; p.r.n., pro re nata.

^a^ “Don’t know” was an answering option for all items included in the table. Therefore, numbers do not sum to group totals.

^b^ Chi-square calculations were substituted with Fisher exact test in this comparison, as values in 2 of the cells in the contingency table were <5.

^a^ Not applicable, as the patients already are treated in a palliative care unit.

## Discussion

This national register study showed that dying patients with both cancer and COVID-19 more often suffered from the breakthrough of breathlessness when cared for in hospitals compared to SPC, while SPC patients had more pain. Symptom assessments and communication about impending death with patients and relatives were significantly less common among patients in hospitals.

### Symptoms and Symptom Assessment

Breathlessness was a common symptom in this study, which is in accordance with other studies among patients with COVID-19^6,10^ and also among patients with COVID-19 and cancer during EOL.^19^ More patients within the hospital group suffered from breathlessness, which indicates that they might have had more severe lung complications from COVID-19. A lower symptom burden during EOL among nursing-home residents with a COVID-19 infection compared to elderly patients in hospitals has previously been shown, which also is an indication that those with more severe COVID-19 have an increased probability of being hospitalized.^24^ Another explanatory factor could be the higher number of comorbidities among those in hospitals, for example, lung and heart disease, which could contribute to more breathlessness. However, in cases of breathlessness as a breakthrough symptom, complete relief was significantly more often achieved in SPC, despite more resources to handle acute respiratory failure in acute hospitals.

As expected, pain was still very common, both in hospitals and in SPC, indicating that the majority of patients had an aggressive cancer disease, as pain is a very common symptom in advanced cancer.^25^ As pain was even more frequently seen among patients in SPC, and this suggests that patient selection is a reasonable explanation for differences in symptom burden. Patients with advanced cancer and complex pain are typically referred to SPC. Patients with advanced cancer who were still living in their own homes but developed acute breathlessness due to COVID-19 were likely to visit emergency departments and subsequently die in the hospital. Moreover, those in hospitals might have deteriorated and died due to their COVID-19 infection, while those in SPC could have already been in EOL before getting a COVID-19 infection, and this difference between patients could have affected the results.

Another aspect regarding symptom relief in hospitals is that patients with advanced cancer and COVID-19 with pronounced needs for symptom relief might not have been referred to SPC due to being contagious. Furthermore, because of the unpredictable course of COVID-19, it might have been difficult to assess which patients would not benefit from further hospital care. A previous study has also shown that lack of consultation of SPC in hospitals decreased the number of patients referred to EOL care in patients with COVID-19 and cancer,^19^ and in our study, only 14% of patients received palliative consultation, which might have decreased referral to SPC.

Symptom assessment and symptom control are key principles of palliative care,^26^ which could explain the significantly higher proportion of patients being assessed for symptoms, and in addition, having complete relief from symptoms in the SPC group. On the other hand, only 50% of patients in SPC were assessed regarding symptoms other than pain, and thus there is a need to increase the symptom assessment during EOL care both in hospitals and in SPC. During the pandemic, studies have emphasized the need to incorporate palliative care into COVID-19 care to enable effective symptom management and also, for example, to increase decision-making regarding nonbeneficial treatments during EOL and bereavement support.^17,27^ As EOL seldom is the main focus in hospitals, inclusion of SPC consultation could be one way of increasing symptom assessment and symptom control by giving recommendations on appropriate medication and care,^28^ especially as consultations were scarce in our study with national coverage.

In addition, to achieve good symptom control, prescription of p.r.n. medications for symptom alleviation is important.^29^ This study showed that most patients had such prescriptions, although the numbers were lower for the hospital group. In concordance with our results, a previous study showed that patients with both cancer and COVID-19 dying in SPC had higher numbers of prescribed medications for symptom control compared to patients dying in hospitals, reflecting the need for palliative care knowledge and routines to control symptoms during EOL care.^19^

### Communication

Previous studies have shown that the relatives’ experience of communication with HCPs during EOL has not been optimal during the pandemic.^9,30^ This study showed that significantly fewer patients and relatives had been informed about the transition to EOL care in hospitals, despite the acute situation. These EOL discussions are experienced as important for relatives,^31^ to prepare for what to expect, to be involved in decision-making during EOL, and also to have the possibility of being present during the dying phase. In addition, good communication seems to be an important factor in the bereavement process and experience of EOL care.^32^ Strengthening SPC consultations in Swedish hospital care could increase the number of EOL discussions and simultaneously improve the quality of care.

Visiting restrictions during the pandemic have profoundly affected the possibility for relatives to visit their loved ones during EOL.^33^ We found that those dying in hospitals were significantly more often dying alone compared to those in SPC, and only one-fourth of those in hospitals had someone from the family present. Similar results have been shown in previous studies,^7^ and these results can be compared with up to 80% having someone present during the dying phase before the pandemic.^23^ In Sweden, there were no enforced quarantines for infected households and thus patients in specialized palliative home care might have had more visits during EOL care compared to inpatient care. As not being alone in the EOL is almost a universal wish,^34^ the possibility to have close ones present^35^ should be highly prioritized and an important lesson to take into account during future pandemics. Also, having the possibility to say goodbye is important for relatives in the bereavement process.^36^

### Strengths and Limitations

This study used data from the SRPC, a national quality register collecting data about the quality of EOL care in Sweden using an established and validated data collection method.^22^ Data about ongoing or previous COVID-19 infections for the reported persons have been collected by the SRPC since the beginning of the pandemic. The official total death toll in Sweden related to COVID-19 was around 16,000 at the end of January 2022, while more than 11,500 cases were registered in the SRPC. During the first 6 months of the pandemic, testing was limited, meaning that some of the included patients in this study had a clinical but not a laboratory-verified diagnosis.

In the register data, information about fluids and enteral tube nutrition during the last 24 h in life is collected with the same item (answered with “Yes,” “No,” or “Don’t know”) and cannot be distinguished from one another. However, based on clinical experience and comparison between SRPC data and medical records (data not published), enteral tube nutrition is seldom used during the last 24 h in Sweden.

## Conclusions

This national register study showed that dying patients with both cancer and COVID-19 more often suffered from the breakthrough of breathlessness when cared for in hospitals compared to SPC, while SPC patients had more pain. When present, symptoms were more often fully alleviated in SPC. Also, symptom assessments and communication about impending death with patients and relatives were significantly less common among patients in hospitals. An improvement, as well as a higher awareness of palliative care routines, may be an important factor for better symptom control and higher quality of EOL care in hospitals.
